# Supplementary material for: Microbiological Properties and Cytotoxicity of PNVCL Hydrogels Containing Flavonoids as Intracanal Medication for Endodontic Therapy
Source: J Funct Biomater. 2022 Dec 17;13(4):305. doi: 10.3390/jfb13040305 (PMC9853322; doi:10.3390/jfb13040305)
Supplement: Supplementary file 1 [file jfb-13-00305-s001.zip › jfb-2050543-supplementary material.pdf]

Table S1. Compounds used in this study: codes, synonyms, chemical structure, empirical formula, and molecular weight.

Compound Code*	Synomyms	Chemical structure	Molecular weight (g/mol)
Ampelopsin (AMP) 42866	(2R,3R)-3,5,7-Trihydroxy-2-(3,4,5-trihydroxyphenyl)-2,3-dihydrochromen-4-one, Ampelopsin, DHM, 3,3′,4′,5,5′,7-Hexahydroxyflavanone, Ampeloptin	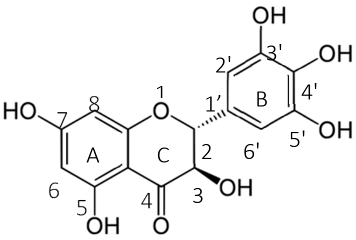	320.25
Isoquercitrin (ISO) #17793	3,3′,4′,5,7-Pentahydroxyflavone 3- β-glucoside, Isoquercitrin	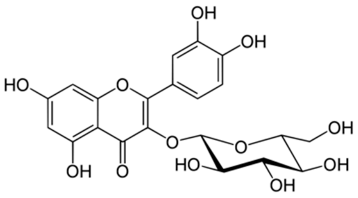	464.38
Rutin (RUT) #R5143	Quercetin-3-rutinoside hydrate, Vitamin P hydrate	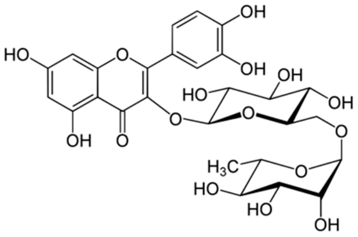	610.52
Chlorexidine digluconate (CHX) #C9394	1,6-Bis(N^5-[p-chlorophenyl]-N^1-biguanido)hexane; 1,1′-Hexamethylenebis(5-[p-chlorophenyl]biguanide)	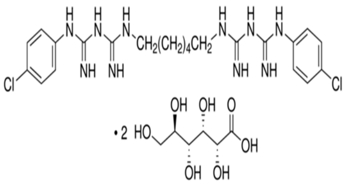	505.44
Calcium hydroxide (CH) #232932	calcium hydrate, lime, hydrated lime, caustic lime, lime hydrate, slaked lime	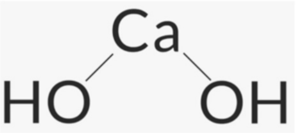	74.09

Poly(N-vinylcaprolactam) (PNVCL)**	1-Ethenylazepan-2-one, 1-Ethenylhexahydro-2*H* -azepin-2-one, 1-Vinylazepan-2-one	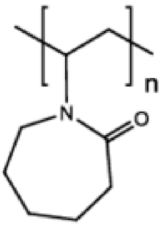	M_n = 25451<br>M_z = 153671

*Codes and information taken from the Sigma-Aldrich company website (https://www.sigmaaldrich.com).

** [1]

1. Sala, R.L.; Kwon, M.Y.; Kim, M.; Gullbrand, S.E.; Henning, E.A.; Mauck, R.L.; Camargo, E.R.; Burdick, J.A. Thermosensitive poly(N-vinylcaprolactam) Injectable hydrogels for cartilage tissue engineering. *Tissue Eng. Part A*. 2017, 23, 935–945.
